# Editorial to the Special Issue: “Dedicated to the 55th Anniversary of G.B. Elyakov Pacific Institute of Bioorganic Chemistry of the Far Eastern Branch of the Russian Academy of Sciences”

**DOI:** 10.3390/molecules26164971

**Published:** 2021-08-17

**Authors:** Pavel S. Dmitrenok

**Affiliations:** G.B. Elyakov Pacific Institute of Bioorganic Chemistry, Far Eastern Branch, Russian Academy of Sciences, Pr. 100 Let Vladivostoku, 159, 690022 Vladivostok, Russia; paveldmt@piboc.dvo.ru; Tel.: +7-423-231-14-30

The G.B. Elyakov Pacific Institute of Bioorganic Chemistry of the Far-Eastern Branch of the Russian Academy of Sciences (PIBOC FEB RAS) was founded in 1964 in Vladivostok in the Far East of Russia. Over many years, we have been carrying out studies on the natural products of both marine and terrestrial origin. In collaboration with many Russian and foreign scientists, we have investigated many hundreds of diverse biomolecules, including steroids and terpenoids, quinoid compounds and alkaloids, polysaccharides and lipids, enzymes and lectins, proteins, and peptides. The Institute has a collection of marine microorganisms (KMM) PIBOC, which includes more than 4000 strains of marine bacteria and more than 1000 strains of marine fungi. The biological activity of natural compounds is also being studied.

This part of the Special Issue is devoted to the investigation of structures and biological activities of low molecular weight secondary metabolites of marine origin. These studies began in the 60s of the last century, but they remain relevant to this day [1]. In the review written by Valentin Stonik and Inna Stonik [2], the information concerning biosynthesis, environmental roles, biological action, and syntheses of marine excitatory amino acids, including kainic acid, domoic acid, dysiherbaine, and neodysiherbaine A, is summarized.

Several articles have described the structural studies of marine secondary metabolites. The structures and absolute configurations of all new compounds have been established by extensive NMR, MS, and ECD analyses, sometimes together with quantum-chemical modeling. Kicha et al. [3] reported about the isolation of four new polyhydroxylated steroids from the Vietnamese starfish *Anthenoides laevigatus*, which is able to inhibit the formation of colonies of human colorectal carcinoma HT-29 and breast cancer MDA-MB-231 cells in non-toxic concentrations. Two compounds have the rare starfish steroid compounds 5β-cholestane skeleton. The studies on a Vietnamese marine sponge *Stelletta* sp. by Kolesnikova et al. [4] led to the isolation of two new isomalabaricane triterpenoids and four new isomalabaricane-derived nor-terpenoids. One of these compounds contains an acetylenic fragment, unprecedented in the isomalabaricane family and extremely rare in other marine sponge terpenoids.

In their research article, Santalova et al. [5] analyzed rare minor oxidized cerebrosides isolated from the extract of the Far-Eastern deep-sea glass sponge *Aulosaccus* sp. Along with NMR spectroscopy and mass spectrometry, GC analysis and chemical transformations were used for structural elucidation of components. The additional instrumental and chemical methods used made it possible, for the first time, to carry out a detailed structural analysis of a complex mixture of glycosphingolipids containing allyl oxygenated monoene acyl chains.

Mishchenko et al. [6] reported on the products formed during the oxidation of Echinochrome A (Ech A). Ech A is one of the main pigments of several species of sea urchins and is registered in the Russian Pharmacopoeia as an active medicinal substance (Histochrome^®^) used in cardiology and ophthalmology. The importance of this work is due to the need to characterize the products formed during the destruction of this pharmaceutical compound and to evaluate the toxic properties of obtaining products.

Sabutsky et al. [7] described the synthesis of new tetracyclic oxathiine-fused quinone-thioglycoside conjugates based on biologically active 1,4-naphthoquinones and 1-mercapto derivatives of per-O-acetyl D-glucose, D-galactose, D-xylose, and L-arabinose. The cytotoxic and antimicrobial activities of the obtained compounds were studied, and the positive effect of heterocyclization with mercaptosugars on the cytotoxic and antimicrobial properties for 1,4-naphthoquinones was shown.

In recent years, studies of the anticancer activity of marine natural compounds have become increasingly important [1,8,9]. Malyarenko et al. [10] investigated the anticancer and radiosensitizing effects of high molecular weight floretols CcPh from the brown algae *Costaria costata* on human colorectal carcinoma cells HCT 116 and HT-29. It was shown that CcPh at non-toxic concentrations suppressed the colony formation of colon cancer cells and significantly increased their sensitivity to low, non-toxic X-ray irradiation, showing a synergistic effect, which can be used to improve the radiation therapy scheme. Kvetkina et al. [11] studied the anticancer activity of the recombinant analog of actinoporin Hct-S3 (rHct-S3) from the sea anemone *Heteractis crispa*. The mechanisms of the anti-migration activity of rHct-S3 and its effect on the programmed death of cancer cells were described. Kaluzhskiy et al. [12], using both a surface plasmon resonance optical biosensor and spectral titration assays, showed that the natural flavonoid luteolin 7,3′-disulfate inhibits the activity of lanosterol 14-alpha demethylase CYP51A1, which may be important for further study of natural flavonoids as cholesterol-lowering and anticancer compounds.

The study of biopolymers of marine origin remains extremely relevant. In order to study the channels of the outer membranes that determine cell permeability, Novikova et al. [13] isolated and characterized the porin MpOmp from the extreme living marine bacterium *Marinomonas primoryensis* KMM 3633^T^. It was concluded that lipid–protein interactions could be a factor that stabilizes the trimeric structure of MpOmp. Bakholdina et al. [14] investigated the effect of cultivation temperatures on the conformational quality of *Yersinia pseudotuberculosis* phospholipase A1 in inclusion bodies (IBs) using green fluorescent protein (GFP) as a folding reporter. Obtained data showed that the GFP-marker could be useful for studying the molecular organization of IBs, their morphology, and localization in *E. coli*, as well as for visualization of IBs interactions with eukaryotic cells.

The study of the genomes of marine bacteria is extremely important, not only for species identification but also for the search for bacteria that are promising sources of unique enzymes and secondary metabolites. Noskova et al. [15] proposed to use the gene of alkaline phosphatase as an additional marker in the description of strains of the genus *Cobetia* due to difficulty in their identification, both in terms of phenotypic parameters and because of the high homology of their 16S rRNA. Bystritskaya et al. [16] investigated general porin regulation in the Far-Eastern strain of *Y. pseudotuberculosis* in response to sublethal concentrations of antibiotics. As a result, the phenotypic heterogeneity of the *Y. pseudotuberculosis* population was found, manifested in the variable expression of the porin gene under the influence of carbenicillin. Chernysheva et al. [17] carried out a detailed study of PL7 alginate lyase in the representatives of the genus *Zobellia*. PL7 was found to belong to subfamilies 3, 5, and 6, undergoing local and horizontal gene transfer and gene duplication processes.

Overall, 14 manuscripts published in the current SI cover almost all aspects of PIBOC research activity in the fields of bioorganic chemistry, biochemistry, organic synthesis of natural compounds, marine microbiology, and genetic engineering, and, we hope, provide interesting new information for scientists working in these fields.
